# Drone imagery dataset for early-season weed classification in maize and tomato crops

**DOI:** 10.1016/j.dib.2024.111203

**Published:** 2024-12-06

**Authors:** Gustavo A. Mesías-Ruiz, José M. Peña, Ana I. de Castro, José Dorado

**Affiliations:** a Institute of Agricultural Sciences, Spanish National Research Council (ICA-CSIC), Serrano 115b, 28006 Madrid, Spain; b Environment and Agronomy Department, National Agricultural and Food Research and Technology Institute (INIA-CSIC), Ctra. Coruña km 7.5, Madrid, 28008 Madrid, Spain

**Keywords:** UAV-imagery, Deep learning, Weed species, Weed mapping, Summer row crops

## Abstract

Identifying weed species at early-growth stages is critical for precision agriculture. Accurate classification at the species-level enables targeted control measures, significantly reducing pesticide use. This paper presents a dataset of RGB images captured with a Sony ILCE-6300L camera mounted on an unmanned aerial vehicle (UAV) flying at an altitude of 11 m above ground level. The dataset covers various agricultural fields in Spain, focusing on two summer crops: maize and tomato. It is designed to enhance early-season weed classification accuracy by including images from two phenological stages. Specifically, the dataset contains 31,002 labeled images from the early-growth stage—maize with four unfolded leaves (BBCH14) and tomato with the first flower bud visible (BBCH501)—as well as 36,556 images from a more advanced-growth stage—maize with seven unfolded leaves (BBCH17) and tomato with the ninth flower bud visible (BBCH509). In maize, the weed species include *Atriplex patula, Chenopodium album, Convolvulus arvensis, Datura ferox, Lolium rigidum, Salsola kali* and *Sorghum halepense*. In tomato, the weed species include *Cyperus rotundus, Portulaca oleracea* and *Solanum nigrum*. The images, stored in JPG format, were labeled in orthomosaic partitions, with each image corresponding to a specific plant species. This dataset is ideally suited for developing advanced deep learning models, such as CNNs and ViTs, for early classification of weed species in maize and tomato crops using UAV imagery. By providing this dataset, we aim to advance UAV-based weed detection and mapping technologies, contributing to precision agriculture with more efficient, accurate tools that promote sustainable and profitable farming practices.

Specifications Table

**Table:** 

Subject	Computer Science. Agricultural Sciences.
Specific subject area	Computer Vision and Pattern Recognition. Agronomy and Crop Science*.*
Type of data	Raw.
Data collection	Images were acquired using a Sony ILCE-6300L camera (Sony Group C Tokyo, Japan) mounted on a UAV quadcopter model md4-1000 (microdrones GmbH, Siegen, Germany) flying at an altitude of 11 m above ground level. This was conducted during the early-season of maize and tomato crops that were naturally infested with weeds, on clear, sunny days during midday hours. Orthophotos were generated and partitioned into 1000 × 1000 pixel images for easier handling using Phyton (Algorithm 1). Experts in weed science then visually identified and labeled the weed species with the labelImg software, and the dataset was stored according to the PASCAL VOC convention (Algorithm 2).
Data source location	Maize: CSIC Experimental Farm La Poveda, located in Arganda del Rey, Madrid, Spain (40°18′57.59″ N, 3°29′22.57″ W; datum WGS84). Tomato: Two commercial fields located in Santa Amalia, Badajoz, Spain (38°59′15.58″N, 6°02′57.71″W and 38°59′40.19″N, 5°57′17.54″W; datum WGS84).
Data accessibility	Repository name: DRONEWEED: DRONE imagery dataset for early-season WEED classification [Data set]; DIGITAL.CSIC Data identification number: 10.20350/digitalCSIC/16559 Direct URL to data: 10.20350/digitalCSIC/16559
Related research article	G.A. Mesías-Ruiz, J.M. Peña, A.I. de Castro, I. Borra-Serrano, J. Dorado, Cognitive computing advancements: Improving precision crop protection through UAV imagery for targeted weed monitoring. Remote Sensing 16 (2024) 3026. 10.3390/rs16163026

## 1 Value of the Data

• DRONEWEED dataset supports machine learning researchers in developing technological solutions to enhance the early classification of weed species affecting maize and tomato productivity.
• DRONEWEED dataset can be used to train, validate and test deep learning models, such as CNNs and ViTs, enhancing th robustness and accuracy of weed classification systems.
• The dataset supports computer vision tasks like multiclass classification, enabling exploration of synthetic image generation (e.g., GANs) for data augmentation and model improvement.
• The dataset includes all possible instances and, to our knowledge, is one of the largest publicly accessible datasets on weed species in maize and tomato crops in Spain.

## 2 Background

DRONEWEED dataset [1] was created to provide an open, accessible, high-quality resource for early-season weed classification using UAV imagery. Accurately labeled datasets are crucial for the development of effective and practical deep learning applications [2]. This dataset, annotated in JPG format, is particularly suited for developing advanced models like Convolutional Neural Networks (CNNs) and Vision Transformers (ViTs) for the multiclass classification of weed species at early-growth stages in maize and tomato crops [3,4]. Other datasets, such as CoFly-WeedDB [5] and DeepWeeds [6] also include various weeds but in other domains and image settings. The CoFly-WeedDB dataset focuses on RGB imagery captured at 5 m above a cotton crop in Greece and is limited to three common weed species (Johnsongrass, Purslane and Field bindweed), while the DeepWeeds dataset focuses on eight relevant weed species from grazing areas in Australia. In contrast, DRONEWEED dataset aims to enhance UAV-based weed classification and mapping in two major agricultural crops, driving precision agriculture in tomato and maize through more efficient, accurate and sustainable farming practices [7].

## 3 Data Description

**Fig. 1:**
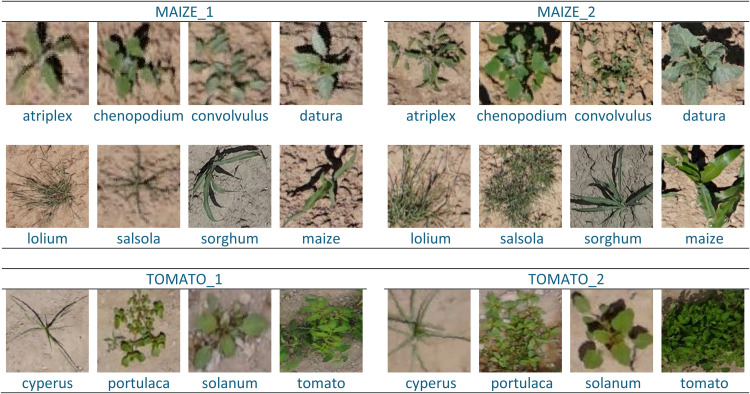
Examples of labeled images in maize crop in BBCH14 (MAIZE_1) and BBCH17 (MAIZE_2) phenological stages, as well as in tomato crop in BBCH501 (TOMATO_1) and BBCH509 (TOMATO_2) phenological stages. Species in MAIZE are: (atriplex) *Atriplex patula*, (chenopodium) *Chenopodium album*, (convolvulus) *Convolvulus arvensis*, (datura) *Datura ferox*, (lolium) *Lolium rigidum*, (salsola) *Salsola kali*, (sorghum) *Sorghum halepense*, and (maize) *Zea mays* L. Species in TOMATO are: (cyperus) *Cyperus rotundus*, (portulaca) *Portulaca oleracea*, (solanum) *Solanum nigrum*, and (tomato) *Solanum lycopersicum* L.

This article presents a comprehensive dataset of imagery featuring weed seedlings and crops, collected during the early-season of maize and tomato in Spain. The dataset comprises 67,558 labeled images, organized into two primary folders: one for maize (ʽMAIZEʼ) and one for tomato (ʽTOMATOʼ). Each folder is further divided into subfolders corresponding to two phenological growth stages: early (e.g. ʽMAIZE_1ʼ and ʽTOMATO_1ʼ) and more advanced (e.g. ʽMAIZE_2ʼ and ʽTOMATO_2ʼ). Within each phenological stage, there are additional subfolders for the weed species associated with each crop, as well as specific folders for the crops themselves. For instance, each ʽMAIZEʼ folder contains eight subfolders: seven for the associated weed species (e.g. ʽMAIZE_#_atriplexʼ for *Atriplex patula* L.; ʽMAIZE_#_chenopodiumʼ for *Chenopodium album* L.; ʽMAIZE_#_convolvulusʼ for *Convolvulus arvensis* L.; ʽMAIZE_#_daturaʼ for *Datura ferox* L.; ʽMAIZE_#_loliumʼ for *Lolium rigidum* Gaud.; ʽMAIZE_#_salsolaʼ for *Salsola kali* L.; and ʽMAIZE_#_sorghumʼ for *Sorghum halepense* (L.) Pers.) and one for maize images (e.g. ʽMAIZE_#_maizeʼ). Similarly, the ʽTOMATOʼ folders contain four subfolders: three for the associated weed species (e.g. ʽTOMATO_#_cyperusʼ for *Cyperus rotundus* L.; ʽTOMATO_#_portulacaʼ for *Portulaca oleracea* L.; and ʽTOMATO_#_solanumʼ for *Solanum nigrum* L.) and one for tomato images (e.g. ʽTOMATO_#_tomatoʼ). These subfolders (organized by crop, phenological stage and species) are provided in ZIP format to facilitate easy downloading. The labeled images, saved in JPG format, have varying resolution dimensions encompassing the entire specimen. Each file is named according to the species (whether weed or crop), the phenological stage (1 for early, 2 for more advanced) and a consecutive number within each subfolder. Fig. 1 shows samples of weed species and crops at both phenological stages.

## 4 Experimental Design, Materials and Methods

### 4.1 Field data collection

**Fig. 2:**
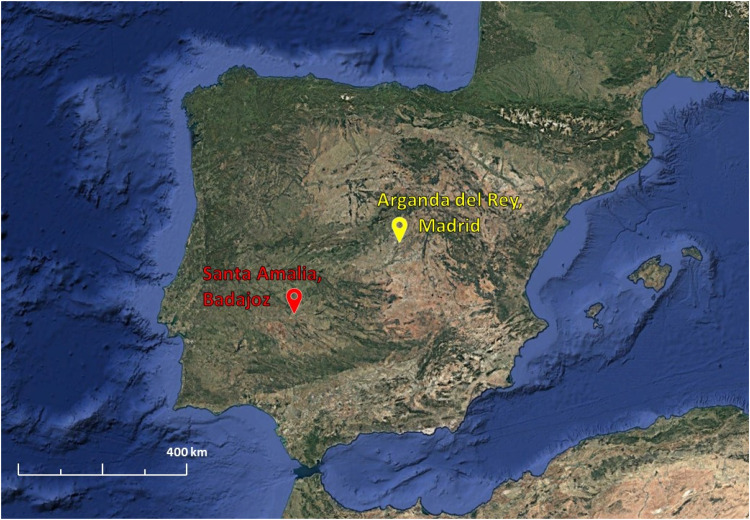
Sampling locations in Spain included the maize crop at the CSIC Experimental Farm in Arganda del Rey, Madrid (yellow label); and the tomato crop in commercial fields in Santa Amalia, Badajoz (red label).(For interpretation of the references to color in this figure legend, the reader is referred to the web version of this article.)

Images were acquired from two distinct locations in Spain (Fig. 2): the CSIC Experimental Farm La Poveda in Arganda del Rey, Madrid (40°18′57.59″N, 3°29′22.57″W; datum WGS84), where maize was cultivated, and two commercial tomato fields in Santa Amalia, Badajoz (38°59′15.58″N, 6°02′57.71″W and 38°59′40.19″N, 5°57′17.54″W; datum WGS84). Sampling was conducted during the crop early-season at two different phenological stages of maize: the four unfolded leaves (BBCH14) on 18/05/2020, and the seven unfolded leaves (BBCH17) on 27/05/2020. Similarly, tomato crops were sampled in two fields with different growth stages: first flower bud visible (BBCH501), and ninth flower bud visible (BBCH509), on 01/06/2021 and 02/06/2021, respectively.

A commercial RGB camera, the Sony ILCE-6300L (Sony Group Corporation, Tokyo, Japan), was mounted on a vertical take-off and landing (VTOL) quadcopter UAV, model md4-1000 (microdrones GmbH, Siegen, Germany), to capture images at a fixed altitude of 11 m above ground level, resulting in a ground sampling distance (GSD) of 0.17 cm per pixel. This high resolution made it possible to capture fine details in the morphological differences of weed species, even in early stages of growth or in conditions of competition with the crop. The resolution setting was calibrated according to the desired geographic coverage and topographic complexity of the study area, considering that the crops analyzed were located in flat and homogeneous areas. A 70 % overlap was maintained both laterally and frontally during image capture, ensuring full coverage of the study area. The camera was equipped with an APS-C Exmor® CMOS sensor (23.5 × 15.6 mm) with 24.2 effective megapixels, producing images with a resolution of 6000 × 3376 pixels. In all cases, image acquisition was performed during zenithal sunlight, which provided optimal illumination conditions by minimizing shadows and maximizing homogeneous illumination on surfaces.

### 4.2 Generation of orthomosaics

The generation of an orthomosaic involved stitching together multiple overlapping UAV images to produce a single high-resolution image that covered the entire area of interest. For maize, 568 and 565 images were used for the BBCH14 and BBCH17 growth stages, respectively, while 895 and 950 images were used for the BBCH501 and BBCH509 growth stages in tomato, respectively, to create orthomosaics of the study fields. This process was carried out using Agisoft PhotoScan software (Agisoft LLC, St. Petersburg, Russia), which utilized information extracted from the RGB channels. Geospatially referenced control points (six to seven, depending on the field) were strategically placed in the experimental fields to ensure accuracy. The orthomosaic corrected distortions caused by the camera angle and varying image perspectives, enabling precise analysis of the objects within the image and facilitating accurate identification and classification of weed species.

### 4.3 Image partitioning

Due to the large size of the generated orthomosaics (e.g. 61,000 × 41,175 pixels for maize at BBCH14, and 72,304 × 34,574 pixels for tomato BBCH501), the images were partitioned into smaller, more manageable sections for individual analysis. This partitioning was carried out using Python and the `rasterio' library (Algorithm 1). The algorithm automatically subdivided the orthomosaics into smaller fragments of 1000 × 1000 pixels, facilitating species recognition and labeling. This approach enhanced processing efficiency and reduced computational complexity for subsequent analyses.

**Algorithm 1:** Orthomosaic partitioning.

1: **Input**: Orthomosaic directory (*orthomosaic*_*directory*), Output directory (*output*_*directory*), Partition size (*m*_*size*) 2: **Output**: Partitioned images 3: function GETCOORDINATESTOPLEFT(*orthomosaic_directory*) 4: **open** the orthomosaic using Rasterio 5: *transf ←* dataset transformation 6: **calculate** the coordinates of the upper left-hand corner 7: **return** coordinates of the upper left corner 8: **end function** 9: function PARTITIONORTOMOSAIC(*orthomosaic_directory, output_directory, m_size*) 10: **create** an empty list for storing the partition data 11: *width, height ←* width and height of the dataset 12 *coordinates (i, j)* ← GETCOORDINATESTOPLEFT (*orthomosaic_directory*) 13: **for each***i* from 0 to *width* with a step of *m*_*size***do** 14: **for each***j* from 0 to *height* with a step of *m*_*size***do** 15: **create** a window for the *partition*[*coordinates*] 16: **read** partition using Rasterio 17: obtain ← *partition[coordinates]* 18: **create** a PIL image from the partition 19: **save** the image in the *output*_*directory* with a name based on the *coordinates* 20: **end for** 21: **end for** 22: **end function**

### 4.4 Species labeling

**Fig. 3:**
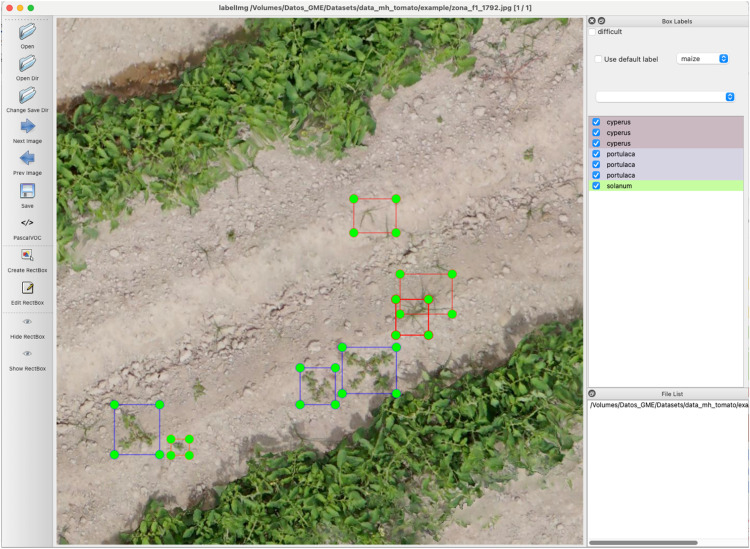
Example of labeling using labelImg software.

**Fig. 4:**
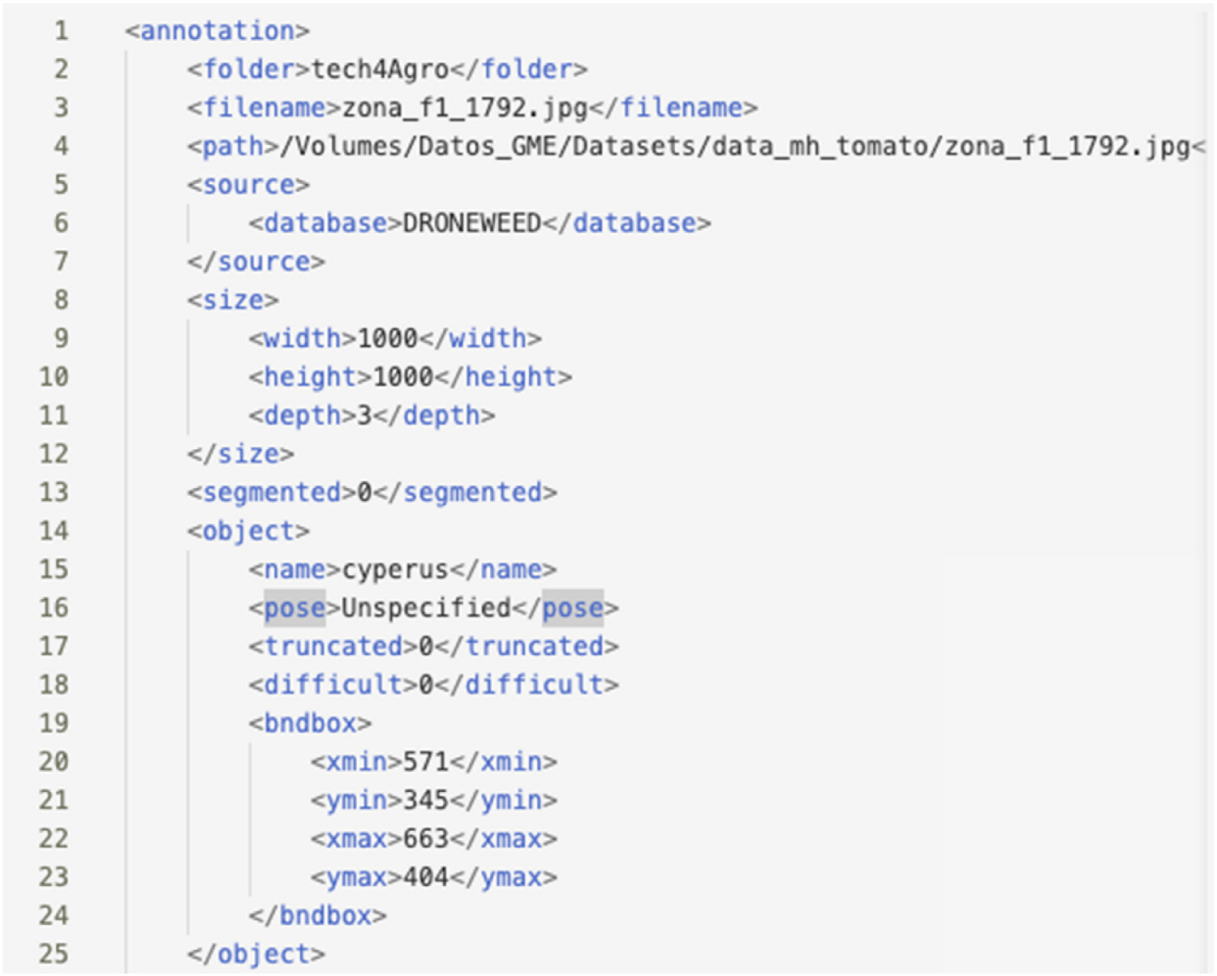
Example of file generated during labeling following the PASCAL VOC convention format.

To ensure reliability and consistency in the labeling process, several key measures were implemented. Prior to UAV image acquisition, a field identification phase was conducted by a team of weed experts, who characterized the weed species present in the maize and tomato crops. Subsequently, image labeling was performed by several experts, which reduced individual bias and improved consistency in species identification. In cases where the identification of weed species in the partitioned images was complex or ambiguous, the experts chose to omit the labeling of these images, thus ensuring the quality and accuracy of the labeled data included in the DRONEWEED dataset. Annotations were made manually. This process involved drawing bounding boxes around each plant and labeling the various objects visible in each subdivided image (i.e. 1000 × 1000 pixels). The labeling was performed using the open-source graphical tool labelImg [8]. Only whole plants were labeled, while those divided by image boundaries were excluded (Fig. 3). The final output was a collection of individual images, each linked to a specific label corresponding to the identified plant species. Each label was saved following the PASCAL VOC convention format (Fig. 4), facilitating structured storage (Algorithm 2) and easy retrieval for use in modeling.

**Algorithm 2:** Cropping and saving labels by species.

1: **Input**: PASCAL VOC files 2: **Output**: processed data stored in different directories 3: **Define**: the directories for each class (species) 4: *species_directories* ← {ʽlolium’ → *path_lolium*, …} 5: **for each** PASCAL VOC file **do** 6: **read** coordinates of bounding box 7: *coordinates* ← [*ymin: ymax, xmin: xmax*] 8: **read** the class value 9: *class* ← *class value* 10: **crop** the image 11: *imagen_cut* ← *imagen*[*coordinates*] 12: **save** the cropped image 13: **if***class* exists in *species_directories***then** 14: save *imagen_cut* in directory *species_directories* 15: **end if** 16: **end for**

### 4.5 Organization and storage of the dataset

The dataset was organized into separate folders based on crop type, phenological stage and weed species (Table 1). This structure organization facilitates easy reuse by researchers aiming to test different deep learning model architectures or develop new image classification techniques for precision agriculture. The dataset compilation process included the following automated steps: i) reading the coordinates of weed bounding boxes from the PASCAL VOC file; ii) extracting weeds from the subdivided images; iii) assigning a unique identifier to each weed corresponding to its specific image section; iv) classifying weed species according to their labels; and v) storing the images in separate directories based on their respective labels. This process was meticulously executed due to the high sensitivity of DL models to both the quality and consistency of the input data. Ensuring that the dataset is diverse, representative, and of sufficient volume is crucial for achieving robust and accurate model performance.

**Table 1:** Number of labeled images for each crop, phenological stage and species included in the database.

	Early-growth stage	More advanced-growth stage
	MAIZE_1 (BBCH14)	MAIZE_2 (BBCH17)
*Atriplex patula*	1000	1459
*Chenopodium album*	1200	2175
*Convolvulus arvensis*	1200	1102
*Datura ferox*	683	589
*Lolium rigidum*	1000	80
*Salsola kali*	1200	1216
*Sorghum halepense*	1600	103
Maize	12,364	24,614
	TOMATO_1 (BBCH501)	TOMATO_2 (BBCH509)
*Cyperus rotundus*	3090	134
*Portulaca oleracea*	1875	177
*Solanum nigrum*	1900	2175
Tomato	3890	2732
Total labels	31,002	36,556

### 4.6 Utility of the dataset

In [3], synthetic images were generated using GANs from the DRONEWEED dataset, obtaining representations of weeds in various morphological configurations and emulating the variations present in advanced stages of growth. This approach not only increased the volume of the dataset, but also introduced new combinations of visual patterns, which improved the accuracy of the models in identifying weeds in complex and varied situations.

### 4.7 Future perspectives

The DRONEWEED dataset was designed to serve as a basis for future research aimed at implementing weed detection models capable of dynamically adapting to seasonal and environmental variations. This will facilitate continuous and accurate monitoring in crop protection using artificial intelligence platforms and implementation of precision agriculture strategies.

## Limitations

The dataset is constrained by the geographical and climatic conditions specific to the study areas in Spain, which may limit the generalizability of the trained models to other regions or crops. Additionally, the dataset primarily focuses on early-growth stages, potentially limiting its applicability for later-growth stages. To overcome this limitation, a useful strategy would be to collect supplementary data from regions with contrasting climatic and soil conditions. For example, integrating data from areas with Mediterranean, semi-arid, or temperate climates could increase the robustness and adaptability of trained models, allowing them to recognize patterns in a wider range of agroecological environments. This would not only improve classification accuracy in other geographic areas, but also enhance the modelʼs ability to adapt to diverse agricultural conditions.

## Ethics Statement

The authors have read and follow the ethical requirements for publication in Data in Brief and confirming that the current work does not involve human subjects, animal experiments, or any data collected from social media platforms.

## CRediT Author Statement

**Gustavo A. Mesías-Ruiz**: Conceptualization, Methodology, Software, Investigation, Data curation, Writing - Original Draft, Writing - Review & Editing. **José M. Peña**: Conceptualization, Methodology, Investigation, Resources, Writing - Review & Editing, Supervision, Project administration, Funding acquisition. **Ana I. de Castro**: Conceptualization, Methodology, Writing - Review & Editing. **José Dorado**: Conceptualization, Methodology, Investigation, Resources, Writing - Original Draft, Writing - Review & Editing, Supervision, Project administration, Funding acquisition*.*

## Data Availability

DIGITAL.CSICDRONEWEED: DRONE imagery dataset for early-season WEED classification (Original data).
